# Protein expression analysis of inflammation-related colon carcinogenesis

**DOI:** 10.4103/1477-3163.51851

**Published:** 2009-06-02

**Authors:** Yumiko Yasui, Takuji Tanaka

**Affiliations:** Department of Oncologic Pathology, Kanazawa Medical University, 1-1 Daigaku, Uchinada, Ishikawa 920-0293, Japan

**Keywords:** Colitis-related carcinogenesis, mice, proteomics analysis

## Abstract

**Background:**

Chronic inflammation is a risk factor for colorectal cancer (CRC) development. The aim of this study was to determine the differences in protein expression between CRC and the surrounding nontumorous colonic tissues in the mice that received azoxymethane (AOM) and dextran sodium sulfate (DSS) using a proteomic analysis.

**Materials and Methods:**

Male ICR mice were given a single intraperitoneal injection of AOM (10 mg/kg body weight), followed by 2% (w/v) DSS in their drinking water for seven days, starting one week after the AOM injection. Colonic adenocarcinoma developed after 20 weeks and a proteomics analysis based on two-dimensional gel electrophoresis and ultraflex TOF/TOF mass spectrometry was conducted in the cancerous and nontumorous tissue specimens.

**Results:**

The proteomic analysis revealed 21 differentially expressed proteins in the cancerous tissues in comparison to the nontumorous tissues. There were five markedly increased proteins (beta-tropomyosin, tropomyosin 1 alpha isoform b, S100 calcium binding protein A9, and an unknown protein) and 16 markedly decreased proteins (Car1 proteins, selenium-binding protein 1, HMG-CoA synthase, thioredoxin 1, 1 Cys peroxiredoxin protein 2, Fcgbp protein, Cytochrome c oxidase, subunit Va, ETHE1 protein, and 7 unknown proteins).

**Conclusions:**

There were 21 differentially expressed proteins in the cancerous tissues of the mice that received AOM and DSS. Their functions include metabolism, the antioxidant system, oxidative stress, mucin production, and inflammation. These findings may provide new insights into the mechanisms of inflammation-related colon carcinogenesis and the establishment of novel therapies and preventative strategies to treat carcinogenesis in the inflamed colon.

## INTRODUCTION

Patients with chronic inflammatory bowel disease (IBD) including ulcerative colitis (UC) are at increased risk of developing colorectal cancer (CRC).[1–4] Indeed, IBD ranks among the top three high-risk conditions for CRC, together with familial adenomatous polyposis (FAP) and hereditary nonpolyposis colorectal cancer (HNPCC).[5] While the latter two hereditary diseases have a well-understood genetic etiology, CRC development in association with IBD appears to be closely relate to chronic inflammation of the large bowel mucosa. Also, IBD-associated colon carcinogenesis can be summarized as an inflammation-dysplasia-carcinoma sequence: hyperplastic lesions in the inflamed mucosa develop CRC through flat dysplasia.[6,7]

An azoxymethane (AOM)/dextran sodium sulfate (DSS) mouse model[8] was used to investigate the changes in global gene expression in the background of inflammation-related colon cancer.[9] A comprehensive DNA microarray analysis revealed that a number of genes altered their expression in the colonic mucosa of mice exposed to AOM/DSS and their expression was significantly increased or decreased in comparison to those found in the mice given AOM or DSS alone.[9] The number of genes with altered expression in the colonic mucosa of the mice that received AOM/DSS at week 5 was greater than that detected at week 10.[9] These genes showing their striking altered expression included *Wif1*, *Plat*, *Myc*, *Plscr2*, *Pparbp*, *Tgfb3*, and *Pparg*.[9]

Comparative proteomic analyses have been used for identifying proteins critical for phenotypic changes that occur during disease development.[10] A reproducible correlation is found between the expression patterns of multiple proteins within epithelial cells and the progression of neoplasms in a variety of tissues, such as the oral cavity,[11] prostate,[12] lung,[13,14] mammary gland,[15] liver,[16] and colon.[17] Yeo *et al*.[18] recently reported that a total of 38 proteins are differentially expressed in colonic tumors and normal mucosa of female C57BL/6 mice that received cycle treatment with DSS. They also stressed the importance of reduced expression of transgelin among the proteins as a biomarker of colitis-related colon carcinogenesis. However, they did not use a colonic carcinogen combined with DSS, rather they used a utilized cycle treatment with DSS to induce CRC in the inflamed colon.

The current study analyzed a number of proteins to isolate and identify tumor specific proteins that might be involved in the development of colitis-related CRC in AOM/DSS model mice[8] by two-dimensional gel electrophoresis to further investigate the protein expression during colitis-associated carcinogenesis.

## MATERIALS AND METHODS

### Animal experiments

#### Animals, chemicals, and diets

Male Crj: CD-1 (ICR) mice (Charles River Japan, Inc., Tokyo) aged five weeks were used in this study. AOM was purchased from Sigma-Aldrich Co. (St. Louis, MO, USA). DSS with a molecular weight of 36,000–50,000 (Cat. No. 160110) was obtained from MP Biomedicals, LLC (Aurora, OH, USA). DSS for the induction of colitis was dissolved in distilled water at a concentration of 2% (w/v). Charles River Formula (CRF)-1 (Oriental Yeast Co., Ltd., Tokyo, Japan) was used as a basal diet throughout the study.

#### Experimental procedure

After arriving, mice were acclimated for seven days with tap water and a pelleted basal diet of CRF-1, ad libitum. They received a single intraperitoneal (i.p.) injection of 10 mg/kg body weight AOM. Starting one week after the AOM injection, the animals were exposed to 2% DSS in the drinking water for seven days, and then were followed without any further treatment until the experiment was done. They were sacrificed by CO_2_ euthanasia at week 20 for the analysis. All mice were maintained at the Kanazawa Medical University Animal Facility according to the Institutional Animal Care Guidelines and were maintained under controlled conditions of humidity (50±10%), light (12/12 hr light/dark cycle), and temperature (23±2°C). The study protocol was approved by the Ethical Committee for animal experimentation of the Kanazawa Medical University.

### Two-dimensional (2-D) gel electrophoresis

#### Chemicals

The sources for chemicals and materials used in the present study were: 3-([3 -Cholamidopropyl]-dimethylammonio)-1-propanesulfonate (CHAPS) from Wako Pure Chemicals (Osaka, Japan), N-decyl-N,N-dimethyl-3-ammonio-1-propane-sulfonate (SB3-10) from Sigma-Aldrich, ampholine from GE Healthcare UK Ltd. (Amersham Place, Little Chalfont, Buckinghamshire HP7 9NA, England), and all other chemicals were purchased from Wako Pure Chemicals.

#### 2-D polyacrylamide gel electrophoresis (PAGE)

Colonic tumors (histologically confirmed as well-differentiated tubular adenocarcinomas) and nontumorous mucosa tissues were collected from the mice that received AOM and DSS and were stored at −80°C prior to use. The frozen tissues were homogenized with five volumes of lysis buffer (5 M urea, 2 M thiourea, 2% CHAPS, 2% SB3-10, 1% dithiothreitol, and 2% ampholine). The protein concentration of these samples was measured using a Protein Assay Kit (Bio-Rad Laboratories). The samples (100 *μ*g) were applied overnight to Immobiline Drystrip (GE Healthcare Bio-Science) by in-gel rehydration.[19,20] The rehydrated gels were then gently dried with tissue paper to remove excess fluid and isoelectric focusing (IEF) was performed in a Multiphor II electrophoresis chamber (GE Healthcare Bio-Science) according to the manufacturer's instructions. Second dimension SDS-PAGE was performed in 9–18% acrylamide gradient gels using an IsoDalt electrophoresis chamber (GE Healthcare Bio-Science). The 2-D gels were stained with SYPRO Ruby (Bio-Rad Laboratories) under the manufacturer's protocols.[21] The SYPRO Ruby stained proteins were detected using the Molecular Imager FX (Bio-Rad Laboratories) and were subjected to in-gel digestion. Image analyses and database management were carried out using the ImageMaster 2D Platinum image analysis software program (GE Healthcare Bio-Science).

#### In-gel digestion and mass spectrometric identification of proteins

Protein spots were excised from the 2-D gels using clean scalpels, and were washed twice with Milli-Q water, and dehydrated in 100% acetonitrile (ACN) until they turned opaque white. The spots were then dried in a vacuum centrifuge, and subsequently rehydrated in 10 μl of digestion solution consisting of 50 mM NH_4_HCO_3_, 5 mM CaCl_2_, 0.01 μg/μl modified sequence-grade trypsin (Promega Co., Ltd.). After incubation for 16 hr at 37°C the digestion was terminated by adding 10 μl of 5% trifluoroacetic acid (TFA). The peptides were extracted three times for 20 mins with 50 μl of 5% TFA, 50% ACN, and the extracts were pooled and dried in a vacuum centrifuge. The dried materials were resuspended with 10 μl of 0.1% TFA. To remove excess salts from the extracts, solid-phase extraction was performed using C_18_ ZipTip (Millipore Co., Ltd.) according to the manufacturer's instructions. The peptides were eluted from the ZipTip by 3 μl of 50% ACN, 0.1% TFA and 1 μl of the eluants were spotted onto a target plate. Then, the spots on the target plate were immediately mixed with 0.5 μl of a matrix solution containing 0.3 mg/ml α-cyano-hydroxycinnamic acid, 33% acetone, 66% ethanol, and were completely air-dried at room temperature. MS and MS/MS spectra were obtained using an Ultraflex TOF/TOF mass spectrometer (Bruker Daltonics Co., Ltd.). An external peptide mixture was used to calibrate the instrument. Identification of proteins was carried out using the MASCOT software (Matrix Science Inc.) with the NCBInr database.

## RESULTS

**Figure 1:**
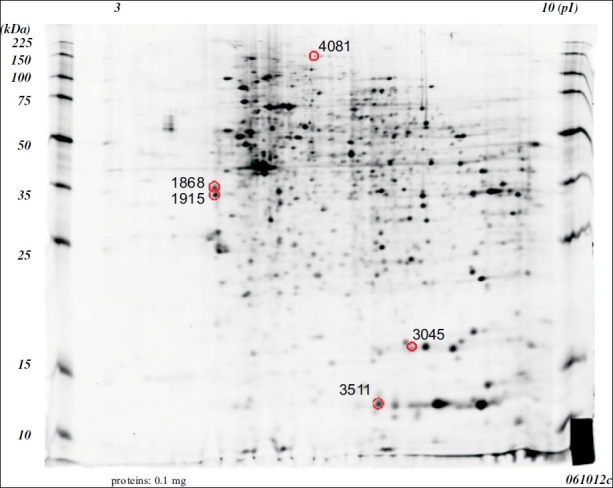
Two dimensional electrophoretic patterns of the whole cell proteins obtained from the cancerous tissues of the mice that received AOM/DSS. The gel was silver stained. The protein spots identified in this study are all circled.

**Figure 2:**
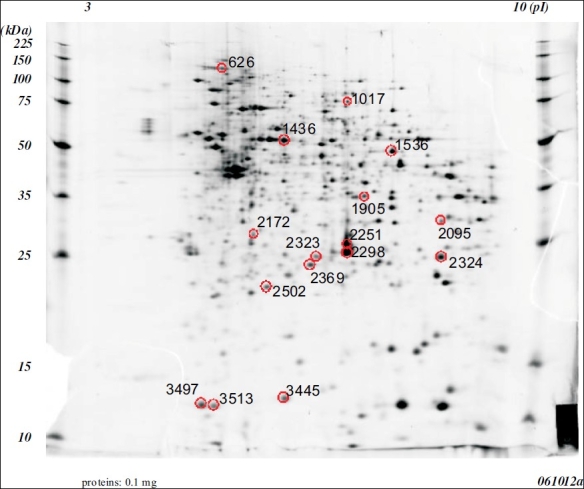
Two dimensional electrophoretic patterns of whole cell proteins obtained from the inflamed colonic mucosa without any tumors in the mice that received AoM/dSS. Gel was silver stained. the protein spots identified in this study are all circle.

A comparative proteomic analysis was conducted on tumors or nontumorous mucosa specimens using 2-DE and MALDI-TOF. We identified 21 spots showing a more than 3.0-fold increase [Figure 1] or decrease [Figure 2] in density in the cancerous tissues. Following trypsin digestion, each spot was analyzed by MALDI-TOF MS, 4 [Table 1] and 9 [Table 2] proteins could be identified.

**Table 1:** Proteins that showed increased expression in the cancerous tissues of mice

Spot no.	Description	Fold change	pl	MW (da)	Accession no.
1915	beta-tropomyosin	8.41	4.61	32982	gil50190
1868	tropomyosin 1, alpha isoform b	7.28	4.71	32746	gil78000190
4081	-	6.47	-	-	N. D.
3511	S100 calcium binding protein A9 (calgranulin B)	3.78	6.64	13211	gil6677837
3045	Peptidylprolyl isomerase A	3.43	7.74	18131	gil53237015

**Table 2:** Proteins that showed decreased expression in the cancerous tissues of mice

Spot no.	Description	Fold change	pl	MW (da)	Accession no.
2172	-	-28.57	-	-	N. D.
2095	-	-18.73	-	-	N. D.
2298	Car1 protein	-17.08	6.44	28370	gil15029975
1436	Selenium-binding protein 1 (56 kDa selenium-binding protein)	-7.39	5.97	52889	gil134259
3445	-	-6.23	-	-	N. D.
2251	Car1 protein	-5.45	6.44	28370	gil15029975
1536	HMG CoA synthase	-5.39	7.69	48384	gil555835
1905	-	-5.14	-	-	N. D.
3497	Thioredoxin 1	-4.77	4.80	12010	gil14789654
2369	1-Cys peroxiredoxin protein 2	-4.49	5.71	24999	gil3789944
2502	-	-3.64	-	-	N. D.
626	Fcgbp protein	-3.45	5.35	72499	gil74179916
3513	Cytochrome c oxidase, subunit Va	-3.38	6.08	16248	gil6680986
2324	-	-3.36	-	-	N. D.
1017	-	-3.14	-	-	N. D.
2323	ETHE1 protein	-3.09	6.78	28234	gil12963539

The proteins with an increased expression in the cancerous tissues were beta-tropomyosin (8.41-fold increase), tropomyosin 1 alpha isoform b (7.28-fold increase), S100 calcium binding protein A9 (calgranulin B, 3.78-fold increase), peptidylprolylisomerase A (3.43-fold increase), and an unknown protein product (6.47-fold increase: Table 1). The proteins with decreased expression in the cancerous tissues were constitutive androstane receptor 1 (Car1) protein (17.08-fold decrease for spot 2298 and 5.45-fold decrease for spot 2251), Selenium-binding protein 1 (SELENBP1, 56 kDa selenium-binding protein, 7.39-fold decrease), 3-hydroxy-3-methylglutaryl coenzyme A (HMG CoA) synthase (5.39-fold decrease), thioredoxin 1 (4.77-fold decrease), 1-cys peroxiredoxin protein 2 (4.49-fold decrease), fcgbp protein (3.45-fold decrease), cytochrome c oxidase, subunit Va (3.38-fold decrease), and ETHE1 protein (3.09-fold decrease: Table 2). Seven other unknown proteins were also identified to show a decreased expression in the cancerous tissues [Table 2].

## DISCUSSION

A comparative proteome analysis of CRC induced by AOM/DSS and the nontumorous mucosa revealed that a total of 21 demonstrated proteins (5 increased and 16 decreased expressions) altered their expression in the cancerous tissues in comparison to the nontumorous tissues. The number of proteins with altered expression was much smaller than genes that showed differential expression patterns in the previous DNA microarray analysis.[9] The proteins that showed altered expression in this study were not consistent with those reported by Yeo *et al*.[18] This discrepancy may be due to the differences of experimental designs between the two studies.

Car1 showed a 17.08-fold decrease for spot 2298 and 5.45-fold decrease for spot 2251 in the cancerous tissue in comparison to the nontumorous tissue. The constitutive androstane receptor (CAR, MB67NR1I3) is a member of the nuclear receptor (NR) superfamily.[22] The expression is most prevalent in the liver, where it mediates the induction of drug and endobiotic metabolism through a mechanism involving the direct regulation of genes encoding biotransformation enzymes.[23,24] Specifically, CAR targets include genes encoding phase I and phase II drug metabolizing functions as well as drug transport genes.[25] The nuclear pregnane X receptor (PXR) and CAR play central roles in protecting the body against environmental xenobiotics.[26] PXR and CAR are activated by a wide range of xenobiotics and regulate cytochrome P450 (CYP) and other genes whose products are involved in the detoxification of these chemicals. Using microarray analyses, a number of CAR-regulated genes have been elucidated,[26] and many of these seem to be directly involved in the metabolism and transport of xenobiotics.[27,28] The expression of many genes involved in xenobiotic/drug metabolism and transport are regulated by at least three nuclear receptors or xenosensors: CAR, PXR, and aryl hydrocarbon receptor. These receptors establish crosstalk with other nuclear receptors or transcription factors controlling signaling pathways that regulate the homeostasis of bile acids, lipids, glucose, inflammation, vitamins, hormones, and others. In the CYP profiles of colon carcinogenesis, significantly higher levels of several CYPs, such as CYP1B1, CYP2S1, CYP2U1, CYP3A5, and CYP51 are present in primary CRC, while CYP3A4 is the most frequently expressed in the normal colon.[29] However, CYP 3A expression is altered in the experimental colitis.[30] While the role of CYPs in IBD is not known, NR is involved in the mediation of inflammatory processes and therefore may play a role in the development of IBD.[31,32] In fact, a significant down-regulation of PXR and the PXR target gene, MDR1, is observed in healthy mucosa adjacent to diseased colonic and terminal ileum of patients with CD and UC.[32] Transgenic mice that do not express the MDR1 gene spontaneously develop colitis under specific pathogen-free conditions and the pathological picture of the colon is quite similar to severe intestinal inflammation observed in IBD.[31] The finding that the expression of CAR1 protein markedly decreased in the cancerous tissue in this study suggests that the decreasing expression of CAR1 protein causes down-regulation of the expression of the metabolism and transport of xenobiotics, such as CYP3A4 or MDR1. The comprehensive DNA microarray analysis using colonic mucosa of AOM/DSS treated and untreated mice,[9] revealed that certain CYP families (CYP2d26, CYP4f16, and CYP2c55) was down-regulated in the colonic mucosa of mice that received AOM/DSS. Thus, CYP may play an important role of the inflammation-related colon carcinogenesis.

HMG-CoA synthase demonstrated a 5.39-fold decrease in the cancerous tissue in this study. HMG-CoA synthase catalyzes a committed step in the pathways for isoprenoid, cholesterol, and ketone body production.[33] Cytosolic HMG-CoA synthase, mainly associated with the production of cholesterol and isoprenoid, is expressed expresses in many tissues and it has a negative feedback mechanism with the accumulation of intracellular cholesterol, which is partly regulated by sterol-regulatory element-binding protein (SREBP),[34] the same as HMG-CoA reductase. Recently, a HMG-CoA reductase inhibitor is reported to activate the transcription of cytosolic HMG-CoA synthase via SREBP.[35] Dietary pitavastatin inhibited AOM/DSS-induced colon carcinogenesis in our previous study,[36] suggesting that HMG-CoA synthase is involved in inflammation-related colon carcinogenesis. On the other hand, mitochondrial HMG-CoA synthase, in which is associated with the production of ketone, is mainly expressed in the liver and intestine. Fasting, cAMP, and fatty acids increase its transcriptional rate, while refeeding and insulin repress the rate.[37] Unlikely cytosolic HMG-CoA synthase, mitochondrial HMG-CoA synthase is regulated by peroxisomal proliferators regulatory element (PPRE), but not SREBP. The regulation of mitochondrial HMG-CoA synthase gene expression by fatty acids is mediated through PPRE, to which peroxisome proliferators activated receptor (PPAR) can bind.[38] PPAR is involved in colorectal oncogenesis.[39] Previously, we reported that dietary administration of ligands for PPARγ and α effectively suppressed the development of colonic epithelial malignancies induced by AOM/DSS in female ICR mice.[40]

The current study showed that Thioredoxin-1 (Trx), a 12kDa protein, was down-regulated by 4.77-fold in the cancerous tissues. This protein is located in the cytoplasm and when translocated into the nucleus has antioxidative and redox-regulating functions. Oxidative stress can be defined as the imbalance between cellular oxidant species production and antioxidant capability. Reactive oxygen species (ROS) are involved in a variety of different cellular processes ranging from apoptosis and necrosis to cell proliferation and carcinogenesis.[4,41] Intracellular Trx regulates DNA binding of several transcription factors including p53, nuclear factor (NF)-κB, and activator protein (AP)-1.[42] In our previous studies, NF-κB is highly expressed in colonic cancer induced by AOM/DSS and certain modulatory agents of its expression inhibit CRC development in the inflamed colon.[41,43,44] In addition, circulating Trx inhibits neutrophil infiltration into the sites of inflammation.[45] These findings suggest that Trx thus plays an important role, not only as an antioxidant and anti-apoptotic molecule, but also as an anti-inflammatory molecule. Therefore, Trx can be a good marker for oxidative stress in various diseases.[46–48] Recently, serum Trx level was reported to be significantly higher in IBD patients and its levels correlated with disease activity.[49] Trx-overexpressing transgenic mice show a decreased severity of colitis in mice treated with DSS.[49] Moreover, the administration of recombinant human Trx decreases the severity of colonic inflammation in interleukin (IL)-10 KO mice.[49] These findings strongly suggest that Trx is involved in the pathophysiology of IBD. Alteration of Trx expression is involved in colitis-related carcinogenesis via regulating DNA binding activity of several transcription factors, including p53, NF-κB, and AP-1 or gene expression associated with inflammation or apoptosis. The other proteins which showed decreased expression in the tumor tissue included Selenium-binding protein 1, HMG CoA synthase, 1-Cys peroxiredoxin protein 2, Fcgbp protein, Cytochrome c oxidase, subunit Va, and ETHE1 protein.

We also observed that the proteins showed highly increased expression in the tumor tissue. The expression of β-tropomyosin and tropomyosin 1 were highly increased by 8.41-fold (spot no. 1915) and 7.28-fold (spot no. 1868), respectively, in the cancer tissue in comparison to the nontumorous tissue. Tropomyosin (TM) is an actin-binding protein, which is localized head to tail along the length of the actin filament and controls cell motility.[50] Although the role of TMs in muscle contraction is well known, their role in nonmuscle cells is less clear. Several lines of evidence suggest that high molecular weight (HMW) TMs encoded by TPM-1 (α-TM) and TPM-2 (β-TM) genes[51] may contribute to the tumor suppressor activity of TGF-β.[52] Bakin *et al*.[52] reported that induction of TMs and stress fibers play an essential role in TGF-β-control of cell motility, and the loss of this TGF-β-response is a critical step in the acquisition of a metastatic phenotype by tumor cells. TMs are involved in pathogenesis of IBD, UC, and CD,[53–55] although the role in colitis-related colon oncogenesis is not known. Other proteins that were highly expressed in the tumor tissue were S100 calcium binding protein A9 and Peptidylprolylisomerase A.

In conclusion, the proteomes of CRC and that of nontumorous mucosa of mice that received AOM and DSS were compared in gels, and differentially expressed proteins were identified by mass spectrometry. A total of 13 proteins from 21 spots were identified by 2-DE and MALDI-TOF MS. Among 13 proteins which showed different expression, CAR1, a member of nuclear receptor superfamily, may play an especially important role in this carcinogenesis model as it showed the most drastic decrease. The study of the protein expression in the tumor tissue and nontumorous mucosa in colitis-associated cancer of a mice model (AOM/DSS model) may help us to find tumor-specific proteins for understanding the pathogenesis of colitis-associated cancer development. Proteomic technologies can thus be used to design rational drugs according to the molecular profile of the cancer cells, and thereby facilitate the development of personalized cancer therapy and prevention.

## CONCLUSIONS

There were 21 proteins differently expressed in the cancerous tissues of mice that received AOM and DSS. Their functions included metabolism, the antioxidant system, oxidative stress, mucin production, and inflammation. This is the first report describing a comprehensive protein expression analysis in an AOM/DSS-induced mouse colon carcinogenesis model. These findings may provide new insights into the mechanisms of inflammation-related colon carcinogenesis and the establishment of novel therapies and preventative strategies against carcinogenesis in inflamed colonic tissue.

## AUTHOR'S PROFILE

**Dr. TAKUJI TANAKA, M.D., Ph.D., FIAC**

1978 - 1983: Assistant Professor, Department of Pathology, Gifu University School of Medicine, Japan

1983 - 1985: Visiting Scientist, Division of Experimental Pathology and Toxicology, Naylor Dana Institute, American Health Foundation, New York, USA

1985 - 1997: Associate Professor, Department of Pathology, Gifu University School of Medicine, Japan

1997 -: Professor and Chairman, Department of Oncologic Pathology, Kanazawa Medical University, Japan

**Specialty and Present Interest:**

Inflammation and Cancer; Cancer chemoprevention by natural products; Biomarkers of carcinogenesis and cancer chemoprevention; Chemical carcinogenesis

**Memberships for Academic Societies:**

Japanese Cancer Association (1976-; Councilor, 2000-)

Japanese Society of Pathology (1976-; Councilor, 1986-)

Japanese Society of Toxicologic Pathology (1986-)

International Academy of Pathology (1987-)

International Academy of Cytology (MIAC, 1987-; FIAC, 1989-)

American Association for Cancer Research (1989-)

European Association for Cancer Research (1991-)

**Editorial Board:**

Journal of Carcinogenesis (Sr. Editor)

Molecular Carcinogenesis

Chemico-Biological Interactions

Asian Pacific Journal of Cancer Prevention

The Open Toxicology Journal

Journal of Toxicologic Pathology

Japanese Journal of Food Chemistry

Journal of Toxicological Sciences (Associate Editor)
